# A Multifunctional Interlayer for Solution Processed High Performance Indium Oxide Transistors

**DOI:** 10.1038/s41598-018-29220-0

**Published:** 2018-07-19

**Authors:** Adrica Kyndiah, Abduleziz Ablat, Seymour Guyot-Reeb, Thorsten Schultz, Fengshuo Zu, Norbert Koch, Patrick Amsalem, Stefano Chiodini, Tugbahan Yilmaz Alic, Yasemin Topal, Mahmut Kus, Lionel Hirsch, Sophie Fasquel, Mamatimin Abbas

**Affiliations:** 1 CNRS, Université Bordeaux, Laboratoire de l’Intégration du Matériau au Système (IMS), UMR 5218, ENSCBP, 16 avenue Pey Berland, 33607 Pessac Cedex, France; 2 School of Physical Science and Technology, Xinjiang University, Urumqi, 830046 People’s Republic of China; 3 Institut für Physik and IRIS Adlershof, Humboldt-Universität zu Berlin, Berlin, Germany; 4 Helmholtz Zentrum Berlin für Materialien und Energie GmbH, 12489 Berlin, Germany; 5 Instituto de Ciencia de Materiales de Madrid, Sor Juana Inés de la Cruz 3, Cantoblanco, 28049 Madrid, Spain; 6 Advanced Technology Research and Application Center, Selcuk University, 42031 Campus, Selçuklu, Konya, Turkey; 7 Present Address: Instituto de Nanociencia de Aragon (INA), Universidad de Zaragoza, 50018 Zaragoza, Spain; 8 Present Address: Gebze Technical University, Institute of Energy Technologies, 41400 Gebze, Kocaeli, Turkey; 9 Present Address: Pamukkale University, Cal Vocational School, 20700 Denizli, Turkey

## Abstract

Multiple functionality of tungsten polyoxometalate (POM) has been achieved applying it as interfacial layer for solution processed high performance In_2_O_3_ thin film transistors, which results in overall improvement of device performance. This approach not only reduces off-current of the device by more than two orders of magnitude, but also leads to a threshold voltage reduction, as well as significantly enhances the mobility through facilitated charge injection from the electrode to the active layer. Such a mechanism has been elucidated through morphological and spectroscopic studies.

## Introduction

Wide band gap metal oxide based thin film transistors (TFT) have been attracting intensive research attention as they have strong application potential in display technology^1^. The most appealing properties of these transistors are their high electrical performance and optical transparency^2–6^. Recently, remarkable progress has been achieved in the development of fabricating such oxide based transistors using low-cost solution processing techniques^7,8^, even on flexible substrates when the curing step was done by a photochemical method^9^.

One such metal oxide semiconductor that has been investigated extensively is indium oxide (In_2_O_3_). In_2_O_3_ transistors intrinsically behave as n-type transistors. They exhibit large electron field effect mobility. However, due to high charge carrier concentration, the Fermi level of In_2_O_3_ is quite close to the conduction band minimum, resulting in high off current in transistor devices^10^. Early efforts had been dedicated to applying binary or ternary compositions using Zn and Ga to improve the electrical characteristics, notably current on/off ratio, but at the expense of reduced mobility compared to single composite In_2_O_3_^10^. Several recent studies have been reported on improving single composite In_2_O_3_ based solution processed TFTs, due to the simplicity of solution formulation, which is appreciated for large scale production. One of them was blend approach, in which it was demonstrated that by doping In_2_O_3_ with an electron rich polymer (polyethylenimine), it was possible to disturb crystallization and control the carrier concentration, so that improved electrical characteristics as well as enhanced electron mobility of about 9 cm^2^ V^−1^s^−1^ can be achieved^11^. Another approach proposed using bilayer In_2_O_3_/ZnO, where doping top layer ZnO with Li metal led to increased electron mobility, albeit with decreased current on/off ratio and negative shift of threshold voltage^12^.

**Figure 1:**
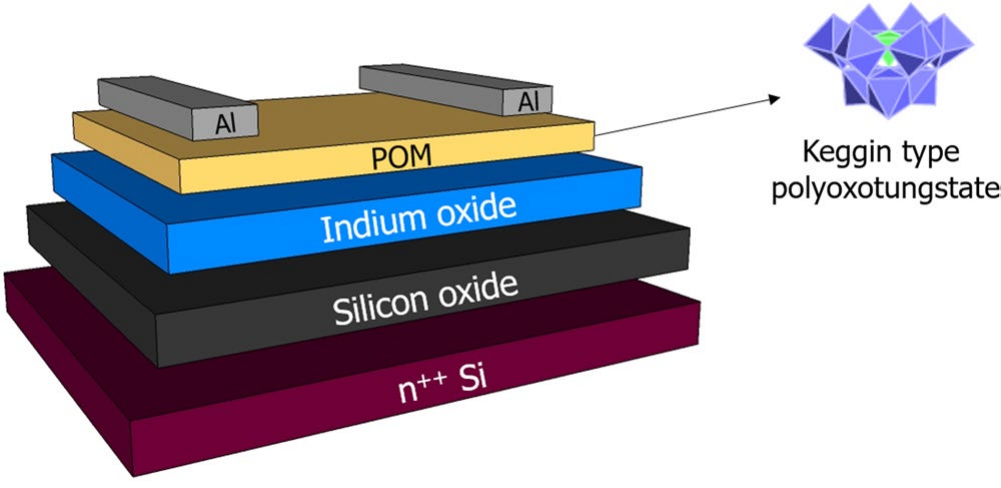
Bottom gate, top contact device architecture of TFTs: Highly doped Si substrate acted as the gate electrode with thermally grown SiO_2_ as dielectric layer. The polyhedral representation of α-K_9_PW_9_O_34_.16H_2_O, which is a trilacunary Keggin type polyoxometalate is also shown. Tetrahedral core represents PO_4_, while each of the nine octahedral represents KWO_6_. Reference device is without POM layer.

In this work, we propose using an interfacial layer with multi-functionality to improve the overall performance of solution processed In_2_O_3_ TFTs. A number of criteria were set for such an interlayer. First, it should have high resistance so that current flow between the source and drain electrode can be minimized. Second, it should have suitable energy levels to facilitate the charge injection from the electrode to the active layer. Third, transparency should be guaranteed for future application of TFTs. Last but not the least, compatible processability is another factor to be taken into account. To this aim, we investigated the role of an interlayer made of kegging type polyoxotungstate (α-K_9_PW_9_O_34_.16H_2_O, POM). POM is an inorganic oxide material that exhibits favorable electron transporting properties and good optical properties due to its optical transparency. It is easily processed by wet chemistry using polar solvents such as water or alcohols^13,14^. Recently, POM was used as an efficient interlayer in organic photovoltaic cells (OPVs). Reports show that OPVs, where POM was inserted as an interlayer, showed enhanced OPV device performances^15^. The same group also incorporated POM as an electron injection layer in their polymer light emitting diodes where an interlayer of POM was inserted between the aluminum cathode and the polymer layer^16^. We present in this work the first successful integration of POM in a transistor device with high performance. Device structure of our TFT and polyhedral representation of POM are shown in Fig. 1.

## Results and Discussion

**Figure 2:**
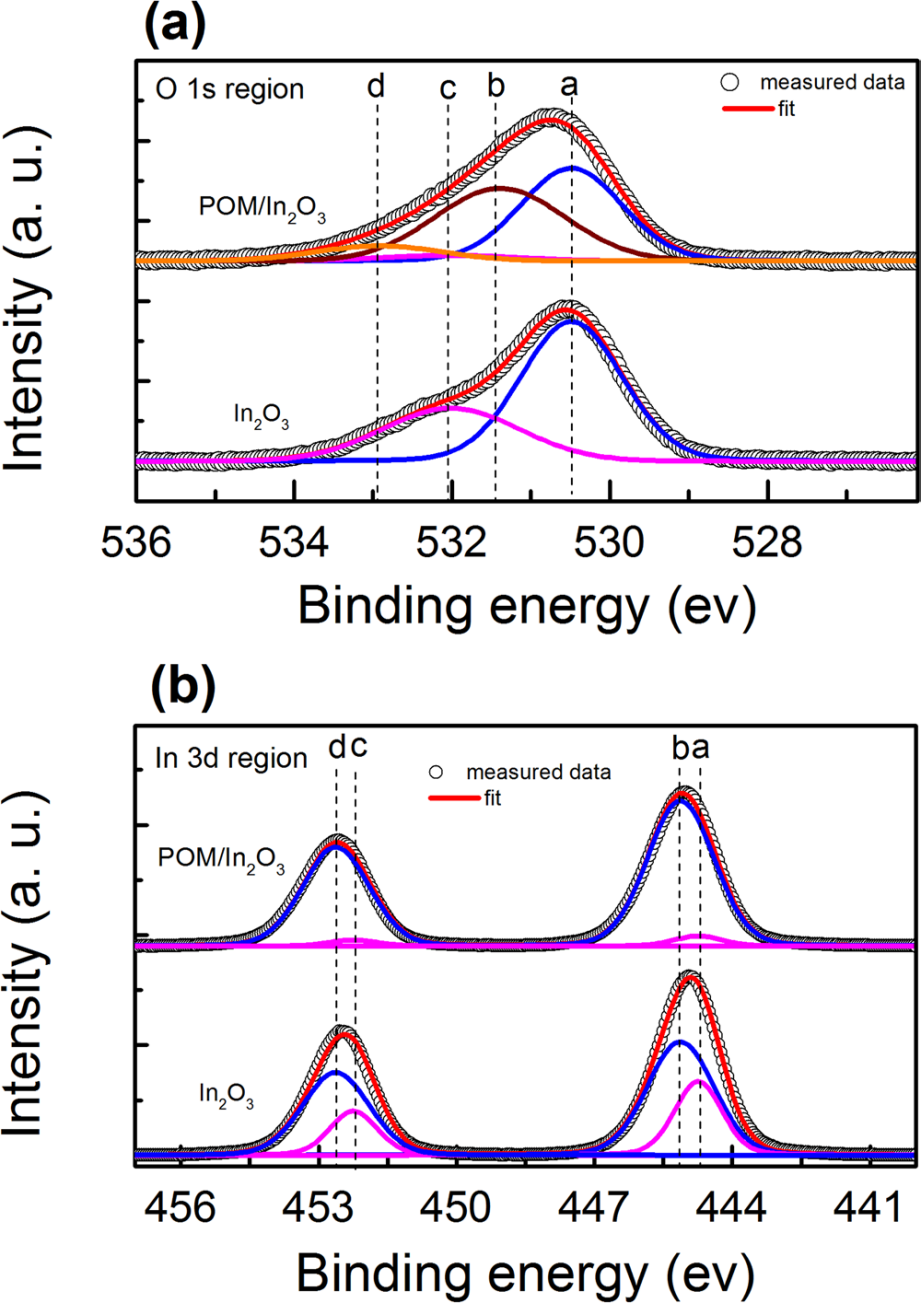
XPS spectra of O 1 s (**a**) and In 3d (**b**) core levels of In_2_O_3_ and POM/In_2_O_3_ films. Fitting was applied after subtracting the background. Dashed lines are the guide to the eye for the positions of the deconvoluted peaks.

In order to investigate the evolution of the surface states following POM layer deposition on the In_2_O_3_, we carried out XPS analysis of O 1 s and In 3d core levels as presented in Fig. 2. O 1 s spectrum of In_2_O_3_ was fitted with two peaks: *a* at 530.5 eV coming from indium-oxygen bonds and *c* at higher binding energy of 532.1 eV from oxygen vacancies^17^. In the case of POM/In_2_O_3_ film, two more peaks appeared: peak *b* at 531.5 eV corresponding to oxygen bonding in KWO_3_ and peak *d* at 532.9 eV related to –OH groups. First notable observation is the presence of high percentage oxygen vacancies up to 33% in In_2_O_3_ film, which is usually the case for solution processed oxide films^17^. Another important observation is that these oxygen vacancies diminished significantly to about 7% when POM layer was deposited on In_2_O_3_ film. Subsequent fitting of In 3d spectra also validates this trend. Lower binding energy peaks of *a* at 444.7 eV (3d_5/2_) and *c* at 452.2 eV (3d_3/2_) can be attributed to the oxygen vacancies, while *b* at 445.1 eV (3d_5/2_) and *d* at 452.6 eV (3d_3/2_) to the indium-oxygen bonds, which clearly confirm the reduction of oxygen vacancies with the deposition of POM layer.

**Figure 3:**
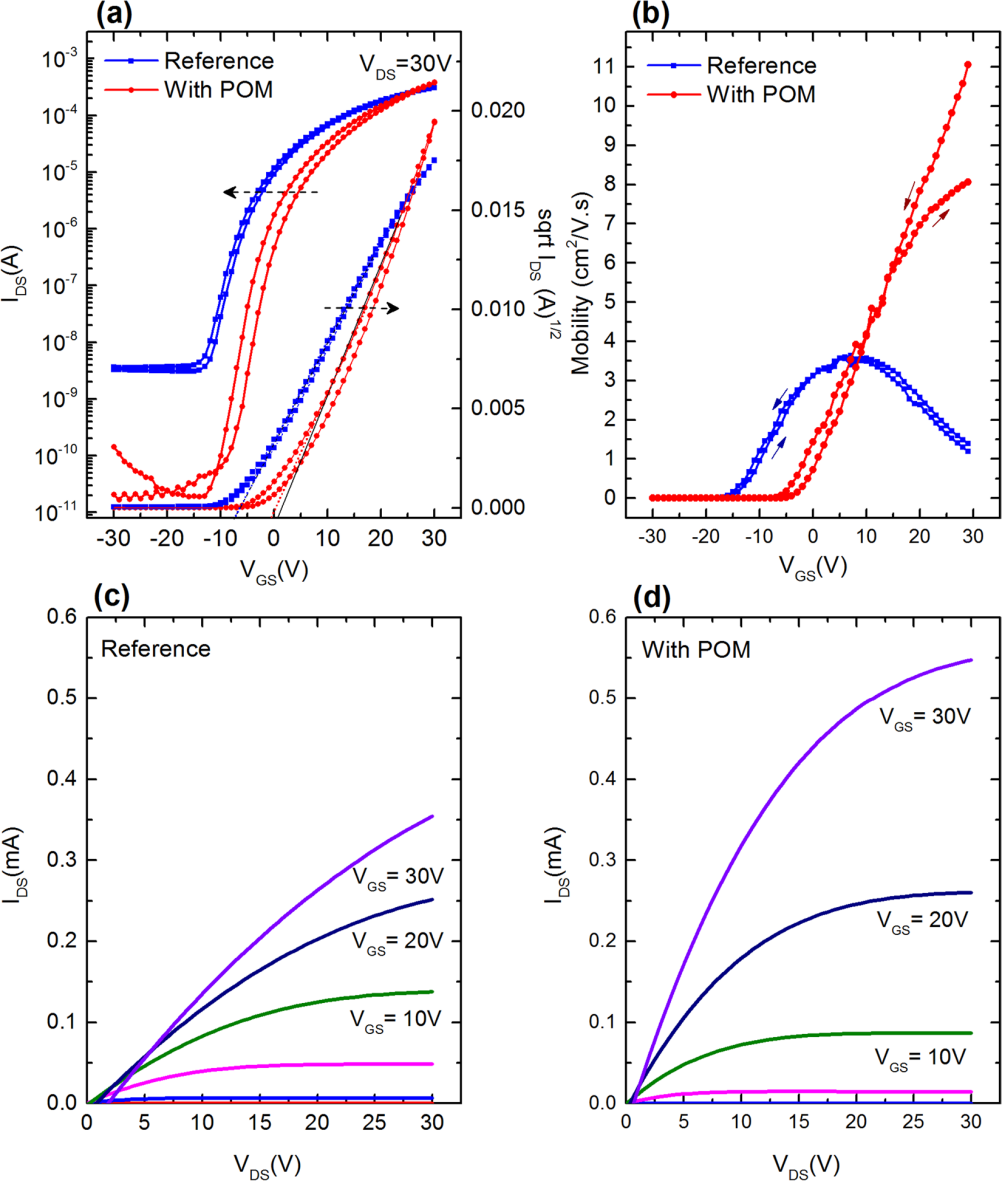
(**a**) Transfer characteristics of TFTs in saturation regime: reference devices (blue curves, square markers) and POM inserted devices (red curves, circle markers); (**b**) Charge carrier mobility curves in saturation regime as a function of V_GS_. The mobility of the reference devices is depicted by the blue curve (square markers) and POM inserted devices by the red curve (circle markers); (**c**) Output characteristics curves of reference devices; (**d**) Output characteristics curves of POM inserted devices.

Figure 3(a) illustrates the typical transistor transfer curves in saturation regime with and without the POM interlayer. As seen from the curves, the device with POM (red curve) clearly shows an enhanced electrical characteristic as compared to the reference devices (blue curve). It should be noted that no channel can be formed in POM, as In_2_O_3_ is in touch with SiO_2_ dielectric, at the interface of which the channel is formed. This is not a bilayer dielectric, as In_2_O_3_ is the semiconducting active layer. The most notable effect is the increase of the current on/off ratio from 10^5^ for reference devices to 10^7^ for POM/In_2_O_3_ devices. High conductivity of the In_2_O_3_ is mainly due to the high concentration of oxygen vacancies, as verified by previous XPS analysis. The addition of POM between the In_2_O_3_ and the Al source-drain electrodes drastically reduces the source-drain current flow in the highly conductive In_2_O_3_ film thus resulting in more than two orders of magnitude lower off current. When the TFT is at off-state, the drain current is controlled by the conductivity of the film. As the resistance of POM is much larger than that of In_2_O_3_, when In_2_O_3_ is separated from the electrodes by POM interlayer, current flow between source and drain electrodes decreases. Moreover, there is also the effect of POM layer in oxygen vacancy reduction on In_2_O_3_ surface as evidenced in XPS analysis above, hence resulting in the lower off-current. One common feature in In_2_O_3_ based TFTs is the negative shift in the threshold voltage, most probably coming from negative local electric field due to the presence of high density of oxygen deficiencies^17^, which is also evident in our reference devices. The threshold voltage is positively shifted back from −10.3 V to 1.5 V with the addition of POM layer, indicating the removal of such a local field at this interface. Subthreshold slope (SS) also improved from 3.1 V/dec to 2.2 V/dec. A slight increase in the hysteresis of POM based devices can be due to the presence of hydroxyl groups which can act as electron traps^18^. Current on/off ratio, threshold voltage and subthreshold slope are not the only parameters that improved by the insertion of POM layer in our devices.

Figure 3(b) shows the derived charge carrier mobility versus gate voltage (V_GS_) plot for both reference and POM/In_2_O_3_ devices. The trend in which the mobility varies as a function of V_GS_ is clearly different. In reference devices, the mobility increases, reaching a maximum of 3.9 cm^2^/Vs for a gate voltage of 10V and then begins to decrease at higher V_GS_. The initial rise in mobility corresponds to initiation of electron injection from the source electrode into the channel. Electrons injected into the channel first fill in bulk and interface trap states and then, as more charges are injected, transport takes place fully along the channel of the transistor leading to further increase in the charge carrier mobility. In amorphous and polycrystalline systems, charge carrier mobility increases with higher carrier concentration^19,20^. However, the decrease in the mobility as V_GS_ further increased can be attributed to strong contact resistance at the electrode and active layer interface^21^. This is due to the fact that when V_GS_ increases, the channel resistance decreases, consequently, the difference between channel and contact resistance becomes smaller, leading to higher potential drop at the contact rather than at the channel. In the case of POM/In_2_O_3_ devices, the mobility increases continuously as a function of V_GS_, revealing that contact resistance is strongly minimized with the addition of POM layer. The maximum mobility of POM/In_2_O_3_ transistors reached 10.8 ± 0.4 cm^2^/Vs, which is significantly higher than that of the reference devices. The output curves for both reference and POM/In_2_O_3_ transistors are shown in Fig. 3(c,d) respectively, with obviously better saturation behavior for the latter device due to largely decreased off current comparing to the reference device. In the linear part, slow onset of output curve in the reference device is an indication of high contact resistance. Contact resistance is drain voltage dependent. At low drain voltage, high contact resistance leads to large potential drop at the contact rather than the channel, thus suppressing the modulated current. TFT parameters are provided in Table 1. When compared to the optimum device reported by Huang *et al*., with similar V_th_ and I_on_/I_off_ ratio, our device with POM shows higher mobility^11^. Although, Khim *et al*. reported similar mobility values for optimized doping concentration, I_on_/I_off_ ratio is two orders of magnitude lower than that of our device^12^.

**Table 1:** TFT performance parameters with and without POM interlayer.

	Mobility (cm^2^/Vs)	V_th_ (V)	SS (V/dec)	I_ON_/I_OFF_
Reference	3.9 ± 0.2	−10.3 ± 0.7	3.1 ± 0.6	10^5^
With POM	10.8 ± 0.4	1.5 ± 1.0	2.2 ± 0.3	10^7^

**Figure 4:**
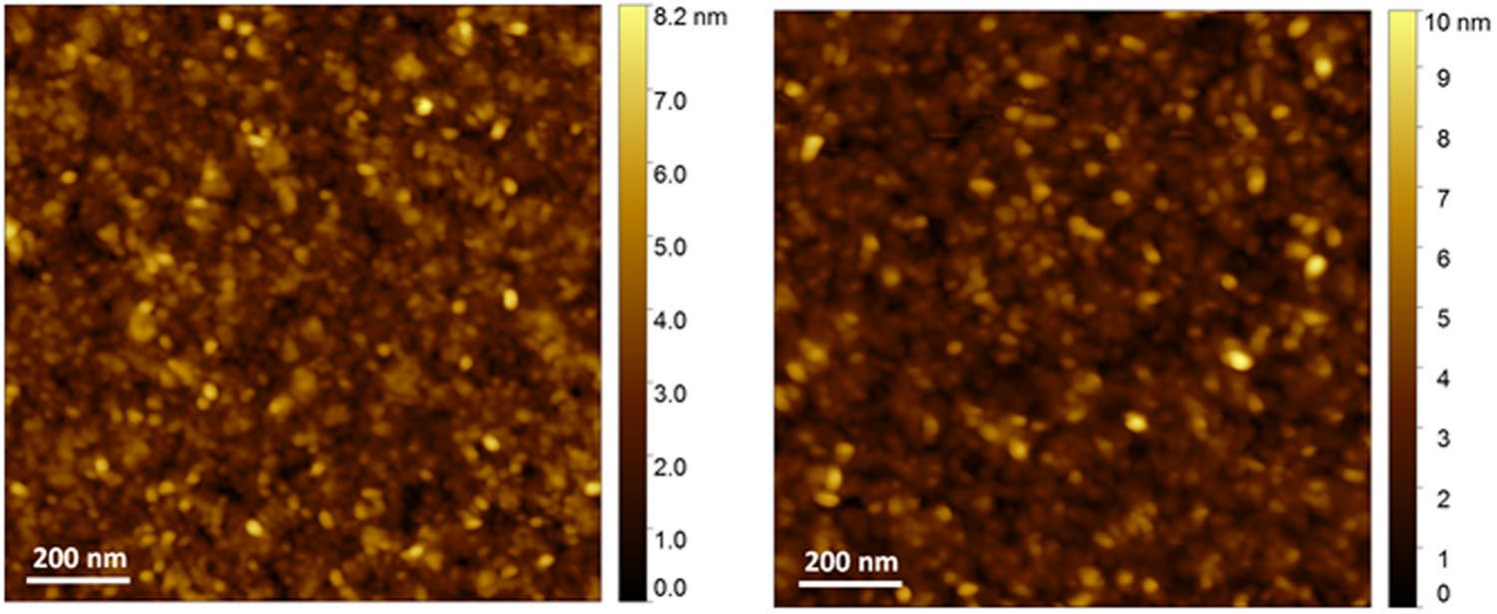
Atomic force microscopy height images of In_2_O_3_ films without (left) and with (right) POM layer.

Such a strong reduction of contact resistance with the addition of POM can originate from either improved surface morphology or facilitated charge injection, as both can have impact on the contact resistance. In order to verify the mechanism, we first carried out atomic force microscopy (AFM)^22^ measurement on the active layer surface before and after POM deposition. We did not observe noticeable differences in the surface morphology of indium oxide by the addition of POM as shown in Fig. 4. The root mean square roughness of the oxide film is (0.88 ± 0.12) nm and after POM deposition is (1.0 ± 0.1) nm, suggesting that the surface morphology is not the factor behind improved performance. Therefore, we further looked at the energy levels at the interface.

**Figure 5:**
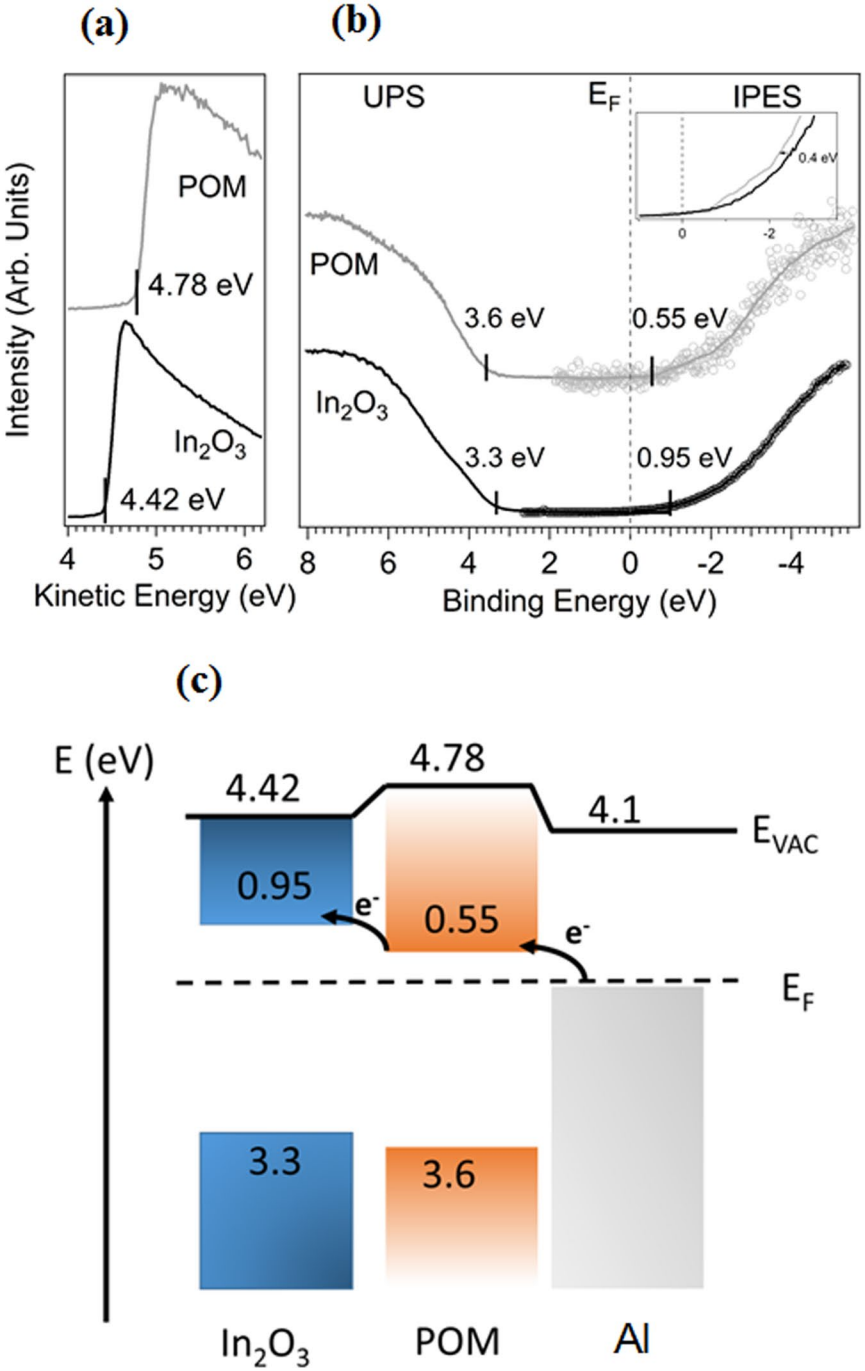
(**a**) Secondary electron cut-off of In_2_O_3_ and POM, yielding work functions of 4.78 eV and 4.42 eV, respectively; (**b**) Combined UPS and IPES measurements of valence and conduction band region for bare In_2_O_3_ and POM. The VBM and CBM of In_2_O_3_ are at 3.3 eV below and 0.95 eV above the Fermi-level, respectively, resulting in a band gap of 4.25 eV. The VBM and CBM of the POM are at 3.6 eV below and 0.55 eV above the Fermi-level, respectively. Inset shows the shift in emission onset. (**c**) Schematic energy level diagram of the In_2_O_3_/POM/Al structure on the basis of UPS/IPES measurements. The insertion of the POM interlayer reduces the electron injection barrier.

We present in Fig. 5 the results of the ultraviolet photoelectron spectroscopy (UPS) and inverse photoemission spectroscopy (IPES) measurements on In_2_O_3_ and POM/In_2_O_3_. From the secondary electron cut-off in Fig. 5(a), the work function can be directly determined, yielding values of 4.42 eV for In_2_O_3_ and 4.78 eV for POM. Our results are in good agreement with literature values for In_2_O_3_ work function, ranging from 4.3 to 5.0 eV^23–25^. The VBM (valence band maximum) and CBM (conduction band minimum) are located 3.3 eV below and 0.95 eV above the Fermi-level (Fig. 5(b)), indicating n-doping of the In_2_O_3_ layer. When POM is deposited on In_2_O_3_, the resulting work function slightly increases to 4.78 eV. The CBM of POM is located very close to the Fermi-level (0.55 eV), whereas the VBM is further away from the Fermi-level (3.6 eV) than the VBM of the In_2_O_3_. These values are in good agreement with previously reported values by Palilis *et al*.^16^. A corresponding energy level diagram is shown in Fig. 5(c). As seen from the diagram, electron injection from Al electrode to In_2_O_3_ through POM is easier than direct injection, which can be the reason for reduced contact resistance at the interface, consequently improved electrical characteristics.

In summary, we investigated the role of tungsten polyoxometalate (POM) as interfacial layer in high performance solution processed In_2_O_3_ thin film transistors. Multiple functionalities were achieved, which led to overall improvement of device parameters. Separation of the electrode from the active layer improved operational threshold voltage, while high resistance coupled with reduced oxygen vacancies decreased the off current more than two orders of magnitude. Suitable energy levels facilitated the electron injection from the electrode to the active layer through POM layer, which was verified by UPS/IPES measurements, and pushed charge carrier mobility over 10 cm^2^/Vs, one of the highest values for single component In_2_O_3_ solution processed TFTs with high current on/off ratio and low threshold voltage. Our results reveal the potential of a single interfacial layer in radically improving device performance of solution processed TFTs.

## Methods

First, α-K_7-x_Na_x_PW_11_O_39_∙14H_2_O was synthesized. In a solution of Na_2_WO_4_∙2H_2_O (181.5 g, 0.550 mol) in 300 mL water, 50 mL of H_3_PO_4_ 1 M and 88 mL of glacial CH_3_COOH were added. The solution was refluxed for one hour, then KCl (60 g, 0.805 mol) was added; the white precipitate which appeared was filtered, washed with water and dried in air. In the next step, 60 mL of K_2_CO_3_ 2 M was added to a solution of 64 g of α-K_7-x_Na_x_PW_11_O_39_∙14H_2_O in 200 mL of water, the white precipitate which appeared was filtered, washed with alcohol and dried in air^26^. The transistors were fabricated on heavily n-doped silicon substrate with a 200 nm thermally grown SiO_2_. Prior to the deposition of In_2_O_3_, the substrates were cleaned by ultra-sonicating them for 10 minutes in acetone, ethanol and isopropanol. After drying the samples, the substrates were then treated with UV ozone for 10 minutes. Indium oxide precursor was prepared by dissolving 30 mg of indium nitrate hydrate (InNO_3_)_3_.xH_2_O in 1 mL of ethylene glycol monomethyl ether. The solution was subjected to rigorous stirring at room temperature for more than 12 hours before using. Oxide layers were deposited from as-prepared precursors by spin coating at 4000 rpm for 60 seconds, followed by thermal-annealing process at 350 °C for 1 hour. Both the deposition and thermal calcination processes were performed in ambient environment. POM solution was prepared by dissolving 1 mg of polyoxotungstate in 1 mL of deionized water. Indium oxide layer was treated by UV ozone for 5 minutes prior to the deposition of POM in order to improve the wettability of the indium oxide surface. POM layer was deposited by spin coating the prepared solution on the indium oxide layer at 5000 rpm for 60 seconds. The sample was annealed on a hot plate at 125 °C for 10 minutes. POM layer thickness is about 10 nm (measured with AFM height profile). Following the deposition of the layers, the substrates were transferred into a nitrogen glove box (O_2_ and H_2_O level <0.1 ppm), where the rest of the fabrication and testing were performed. For the source and drain contacts, 15 nm of aluminum was thermally evaporated using the electron beam deposition technique. The shadow mask used for aluminum deposition defines the source and drain channel length of 50 µm and channel width of 500 µm. The device structure used in this study with a bottom gate, top contact configuration is depicted in Fig. 1 together with the polyhedral representation of POM. The transistors were measured in the glove box using Keithley 4200 semiconductor analyzer system. Source-drain current I_DS_ measurement was performed in saturation regime by sweeping the source gate voltage V_GS_ from −30V to +30 V, keeping the source grounded and source-drain voltage V_DS_ at +30 V. The saturation mobility was derived by taking the derivative of I_DS_ vs V_GS_. The ultraviolet photoelectron spectroscopy (UPS) measurements were conducted at Humboldt-University, using a He discharge lamp (21.2 eV of excitation energy), a SPECS Phoibos 100 hemispherical energy analyzer (resolution 150 meV) and a base pressure of 10^−10^ mbar. The secondary electron cut-off (SECO) was measured with the sample bias of −10V to clear the analyzer work function. The work function (WF) and valence band maximum (VBM) were determined by a linear extrapolation of the SECO and the valence band onset, respectively. Inverse photoelectron spectroscopy (IPES) measurements were performed in the isochromat mode using incident electron energy of 5–13 eV and a NaCl-coated photocathode. The conduction band minimum (CBM) was determined analogously to the VBM. XPS measurements were carried out at ElorprintTec platform in Bordeaux using monochromatic Al Kα source. The spectrometer chamber is equipped with a SPECS Phoibos 100 hemispherical energy analyzer. The AFM images were obtained using a commercial Cypher AFM (Asylum Research, Oxford Instruments). The used AFM cantilever was a PPP-NCHAuD (Nanosensors) with a nominal spring constant *k* = 42 N/m and a resonance frequency *f *≈ 270 kHz. The AFM imaging was performed in air (Q ≈ 600), at a scan rate of about 1 Hz and 512 pixels per line. The roughness measurements were done by Gwyddion, taking the root mean square of the height profile.
